# Analyzing Longitudinal wb-MRI Data and Clinical Course in a Cohort of Former Smoldering Multiple Myeloma Patients: Connections between MRI Findings and Clinical Progression Patterns

**DOI:** 10.3390/cancers13050961

**Published:** 2021-02-25

**Authors:** Markus Wennmann, Thomas Hielscher, Laurent Kintzelé, Bjoern H. Menze, Georg Langs, Maximilian Merz, Sandra Sauer, Hans-Ulrich Kauczor, Heinz-Peter Schlemmer, Stefan Delorme, Hartmut Goldschmidt, Niels Weinhold, Jens Hillengass, Marc-André Weber

**Affiliations:** 1 Division of Radiology, German Cancer Research Center (DKFZ), Im Neuenheimer Feld 280, 69120 Heidelberg, Germany; h.schlemmer@dkfz.de (H.-P.S.); s.delorme@dkfz.de (S.D.); 2 Diagnostic and Interventional Radiology, University Hospital Heidelberg, Im Neuenheimer Feld 110, 69120 Heidelberg, Germany; laurent.kintzele@med.uni-heidelberg.de (L.K.); hans-ulrich.kauczor@med.uni-heidelberg.de (H.-U.K.); marc-andre.weber@med.uni-rostock.de (M.-A.W.); 3 Division of Biostatistics, German Cancer Research Center (DKFZ), Im Neuenheimer Feld 280, 69120 Heidelberg, Germany; t.hielscher@dkfz-heidelberg.de; 4 Department of Computer Science, Technical University of Munich, Boltzmannstrasse 3, 85748 Garching, Germany; bjoern.menze@tum.de; 5 Department of Biomedical Imaging and Image-Guided Therapy, Computational Imaging Research Laboratory, Medical University of Vienna, Währinger Gürtel 18-20, 1090 Vienna, Austria; georg.langs@meduniwien.ac.at; 6 Department of Medicine V, Multiple Myeloma Section, University Hospital Heidelberg, Im Neuenheimer Feld 410, 69120 Heidelberg, Germany; maximilian.merz@med.uni-heidelberg.de (M.M.); sandra.sauer@med.uni-heidelberg.de (S.S.); hartmut.goldschmidt@med.uni-heidelberg.de (H.G.); niels.weinhold@med.uni-heidelberg.de (N.W.); 7 National Center for Tumor Diseases (NCT), University Hospital Heidelberg, Im Neuenheimer Feld 410, 69120 Heidelberg, Germany; 8 Department of Medicine, Roswell Park Comprehensive Cancer Center, Elm and Carlton Streets, Buffalo, NY 14263, USA; jens.hillengass@roswellpark.org; 9 Institute of Diagnostic and Interventional Radiology, Paediatric Radiology and Neuroradiology, University Medical Centre Rostock, Ernst-Heydemann-Str. 6, 18057 Rostock, Germany

**Keywords:** smoldering multiple myeloma, whole-body MRI, MRI, focal lesion, CT, osteolytic lesion, diffuse infiltration, anemia, prognostic assessment, progression pattern

## Abstract

**Simple Summary:**

Smoldering multiple myeloma is a heterogenous precursor state of multiple myeloma—both the time until progression and the cause of progression vary within this subgroup. While the number of focal lesions in whole-body MRI (magnetic resonance imaging) is known to be of prognostic value, we investigated whether additional parameters like the size or growth dynamics of focal lesions or precisely quantified diffuse infiltration are connected to risk and cause of progression. We found that both the size and dynamics of focal lesions correlate with progression to multiple myeloma and with existence/appearance of corresponding osteolytic lesions in near-term computed tomography (CT) scans. Furthermore, a score combining intensity and extent of diffuse infiltration showed connections with risk of progression and decrease in hemoglobin. We conclude that these parameters from whole-body MRI can help to predict the clinical course of disease in smoldering multiple myeloma (SMM) patients, with implications for diagnostic follow-up and possibly more specified therapeutic approaches in the future.

**Abstract:**

The purpose of this study was to analyze size and growth dynamics of focal lesions (FL) as well as to quantify diffuse infiltration (DI) in untreated smoldering multiple myeloma (SMM) patients and correlate those MRI features with timepoint and cause of progression. We investigated 199 whole-body magnetic resonance imaging (wb-MRI) scans originating from longitudinal imaging of 60 SMM patients and 39 computed tomography (CT) scans for corresponding osteolytic lesions (OL) in 17 patients. All FLs >5 mm were manually segmented to quantify volume and growth dynamics, and DI was scored, rating four compartments separately in T1- and fat-saturated T2-weighted images. The majority of patients with at least two FLs showed substantial spatial heterogeneity in growth dynamics. The volume of the largest FL (*p* = 0.001, c-index 0.72), the speed of growth of the fastest growing FL (*p* = 0.003, c-index 0.75), the DI score (DIS, *p* = 0.014, c-index 0.67), and its dynamic over time (DIS dynamic, *p* < 0.001, c-index 0.67) all significantly correlated with the time to progression. Size and growth dynamics of FLs correlated significantly with presence/appearance of OL in CT within 2 years after the respective MRI assessment (*p* = 0.016 and *p* = 0.022). DIS correlated with decrease of hemoglobin (*p* < 0.001). In conclusion, size and growth dynamics of FLs correlate with prognosis and local bone destruction. Connections between MRI findings and progression patterns (fast growing FL—OL; DIS—hemoglobin decrease) might enable more precise diagnostic and therapeutic approaches for SMM patients in the future.

## 1. Introduction

Monoclonal plasma cell disorders originate from clonal plasma cells. They comprise the asymptomatic precursor states monoclonal gammopathy of unknown significance (MGUS) and smoldering multiple myeloma (SMM)—which do not receive treatment—and multiple myeloma (MM), which is defined by showing end organ damage defined by CRAB-criteria (Calcium elevation, Renal insufficiency, Anemia, Bone lesion) or biomarkers of malignancy which indicate high risk of developing end-organ damage within two years, and consequently receive systemic therapy. Despite being one disease, monoclonal plasma cell disorders show marked heterogeneity regarding genomics, phenotype, and prognosis. The genetic architecture is heterogenous on inter- [1,2] and intra- [3] patient level. Symptoms of MM patients vary from bone pain or pathological fractures over renal failure and anemia to calcium elevation and immune deficiency. Finally, even within a single stage, time to progression various enormously; in SMM, 20% of patients become symptomatic within 2 years, while one third does not progress to MM within a decade [4].

The heterogeneity regarding time to progression in SMM has been addressed in two steps: Firstly, by describing several risk factors which enable the isolation of high-risk SMM patients from magnetic resonance imaging (MRI) [5,6,7,8], blood tests [9,10,11,12], and bone marrow biopsy [10,13]. Secondly, these high-risk SMM patients who face imminent progression were upstaged to MM and are now offered systemic therapy [14], as treatment in high-risk SMM has been shown to improve the outcome [15,16,17].

While having >1 focal lesion (FL) in MRI is now used to identify former high-risk SMM patients and upstage them to MM [14], whole-body magnetic resonance imaging (wb-MRI) offers a huge amount of information beyond number of FLs, such as, for example, size or growth dynamic of each FL or extension and local severity of diffuse infiltration. Given the heterogeneity of the disease as described above, in this work we evaluated whether this additional information from wb-MRI could be used to better understand the development of the disease, both regarding when and how progression occurs. Therefore, we studied progression patterns in a cohort of former SMM patients with longitudinal wb-MRI follow-up and without systemic therapy regarding the following aspects.

Firstly, a recent study showed that genetics can differ between tumor sites within one patient and that the diameter of an FL correlates with advancement of local tumor biology at the respective site in MM patients [3]. Given that different genetics might lead to different growth dynamics of FLs, we investigated whether spatial growth heterogeneity can be observed in untreated SMM patients.

Secondly, another recent study demonstrated that the size of FLs besides their number is of prognostic value in symptomatic MM [18]. Consequently, we investigated whether size of an individual FL is of prognostic relevance in SMM. The overall dynamics of MRI findings has also been shown to be a relevant risk factor in SMM [19], and therefore we investigated whether the speed of growth of individual FLs is of prognostic relevance for progression. Additionally, we performed a subgroup analysis on SMM patients with 0 or 1 FL to investigate whether size or growth of a single FL is of value for risk stratification in SMM according to the updated disease definition [14].

Thirdly, a limitation of currently available risk models is that they stratify patients in general high-risk vs. low-risk groups, despite the fact that different kinds of end organ damage—osteolysis, renal insufficiency, anemia, and calcium elevation—appear in MM. These different events might resemble different underlying progression patterns and might be targets for more specified therapeutic approaches. Osteolytic lesions (OL) are the reason for progression to MM in 76% of SMM patients [19], and around 90% of MM patients develop OL throughout the course of their disease [20], making OL the main factor for morbidity in MM. Consequently, a biomarker to predict appearance of OL would be of utmost clinical value. Imaging might have the potential to provide this biomarker. Multiple studies have compared the diagnostic performance between MRI and computed tomography (CT)/X-rays to detect bone involvement in MM [21,22], but no association between FL size or growth and presence/appearance of corresponding OL has yet been reported. An association between diffuse infiltration and severe anemia has been reported in MM [23,24], but the significance of diffuse infiltration in asymptomatic MM has been inconclusive [5,6,19].

With the current state of research and the clinical questions as described above, we used a cohort of SMM patients with longitudinal wb-MRI and long-term clinical follow-up, which allowed the analysis of the progression patterns until end organ damage occurred without the distorting effect of administrated systemic therapy, in order to investigate: (1) if local growth dynamics differ between different FLs in untreated SMM patients; (2) if local size or growth dynamics of FLs is of prognostic value in SMM; (3) if there are connections between MR imaging findings and clinical progression patterns, specifically (3a) between size or growth dynamics of FLs and existence/appearance of OL, and (3b) between diffuse infiltration and development of anemia.

## 2. Patients and Methods

### 2.1. Patients

This study comprised 199 wb-MRIs originating from follow-up wb-MR-imaging of 60 patients diagnosed with SMM according to the definition of the International Myeloma Working Group (IMWG) from 2003 [25]. While patients with indication for systemic therapy were excluded from this analysis, 8 patients who had received local radiation therapy due to a concomitant solitary plasma cell tumor and then entered clinical and imaging follow-up of SMM patients were included in this analysis. All patients received the initial wb-MRI between 2004 and 2011 at our center and underwent MRI follow-up with, on average, 3.3 wb-MRIs with a median time between consecutive MRIs of 13 months. Clinical and imaging follow-up were concluded on 1 January, 2015 due to the updated disease definition at that time [14]. All patients were followed clinically at least every 3 to 6 months until end of observation. Thirty-seven patients were male. At baseline, patients had a median age of 55 years, a median plasma cell infiltration in the bone marrow of 12.5%, and a median serum m-protein of 18 g/L. Thirty-nine patients had no FLs at baseline, while 21 patients showed at least one (mean: 3.5) FL at baseline. The median clinical follow-up was 7 years and 6 months. Twenty-four patients progressed by formal fulfilment of CRAB-criteria [25], one progressed by development of plasma cell leukemia, and four progressed to Salmon and Durie Stage II, which according to the guidelines at that time was considered to be an indication for systemic therapy [26]. One patient received local radiation therapy and was excluded from further analysis, and 2 patients died unrelated to MM. All other patients were followed up for at least 3 years after initial wb-MRI. Patients did not receive prophylactic treatment with bisphosphonates due to their diagnosis with SMM. This study was conducted according to the guidelines of the Declaration of Helsinki and was approved by the Institutional Ethics Committee of the Medical Faculty of the Heidelberg University, Germany with waiver of informed consent (S-511/2016).

### 2.2. Imaging

All wb-MRIs analyzed in this study were acquired by one of two identical 1.5 Tesla MRI systems (Magnetom Avanto, Siemens Healthineers, Erlangen, Germany) and phased-array, body-matrix surface coils of the same manufacturer. Patient positioning and specifics on the sequences for wb-MR imaging at our institutions at that time have been published before [27,28,29]. For analysis of presence/occurrence of corresponding OL to each FL, the imaging database was searched for all CT/ positron emission tomography (PET)-CT imaging available up to 2 years after any MRI examination for this cohort. CTs/PET-CTs after therapy initiation were excluded. It is known that CT detects considerably more OL than a skeletal survey [30] and even revealed OL in 22% of SMM patients who had not shown any OL in conventional skeletal surveys [31]. We consider the superiority of CT over X-ray especially relevant when explicitly searching for small, early OLs (>5 mm in diameter) which represent the initial damage to the mineralized bone, as detected at the time of progression in an imaging follow-up cohort as investigated in this study. As a compromise between size of the dataset and sensitivity for detection of OLs, we therefore restricted the correlation of FLs to corresponding OL to the CT/PET-CT dataset. X-ray imaging, which was the standard of care before 2008 at our institution, was not included. In total, 36 CTs and the CT-component of 3 PET-CTs from 17 patients were included in the analysis for presence/occurrence of OLs corresponding to FLs. Twenty-eight CTs were performed with the specific myeloma/skeletal low-dose wb-CT protocol with the scan parameters as reported before [32]. The remaining 8 CTs included thoracic, neck-to-pelvic, or spinal CT scans, which were present in our picture archiving and communication system and had different scan parameters.

### 2.3. Image and Data Analysis

For assessment of FL volumes, all FLs >5 mm were manually segmented over the complete observation period as described before [29]. The speed of growth was calculated as the ratio of the difference of FL volumes between consecutive exams and the time between those exams, representing a linear growth model which has shown to outperform the exponential growth model in growth of FLs in SMM [33]. For every wb-MRI, the volume of the largest FL, the speed of growth of the largest FL, and the speed of growth of the fastest growing FL were determined. The total tumor volume (TTV) and its dynamic refer to the systemic tumor load (sum of TV of all FL) and its development over time as presented before [29]. For quantification of diffuse infiltration, 4 compartments (humeri, spine, pelvis, and femora) were rated separately in both T1-weighted turbo-spin-echo (tse) and T2-weighted stir (short tau inversion recovery) images, categorizing the marrow intensity changes due to hypercellularity into three classes: none to mild (high signal intensity in T1w tse and low signal intensity in T2w stir): 0 points; moderate (intermediate signal intensities in T1w tse and T2w stir): 1 point; severe (T1w tse signal approximates intervertebral discs/muscle; markedly hyperintense signal in T2w stir): 2 points [34]. These were then added up for the diffuse infiltration score (DIS). The dynamic of the DIS was calculated as the difference in DIS between consecutive exams divided by the elapsed time. Patients with reported history of smoking [35] or earlier radiation therapy affecting the bone marrow [36] were excluded from DIS analysis (*n* = 5 and *n* = 9), as these are known to cause changes to the bone marrow. To investigate a possible correlation between FL characteristics and existence/appearance of a corresponding osteolytic lesion in the respective position, the exact position in the contemporaneous CT for each FL was checked for destruction of mineralized bone ≥5 mm in diameter as defined before [14,37]. To investigate how our findings translate to patients which are considered as SMM according to the updated disease definition [14], we performed a set of subgroup analyses. For the analysis of the heterogeneity of growth dynamic, only patients with concomitant plasma cell tumor were excluded, as at least 2 FL need to be present to investigate spatial growth heterogeneity, exclusion of patients with >1 FL was not applicable. For all other analyses, a subgroup analysis was performed excluding both patients with previously treated concomitant plasma cell tumors and excluding MRI follow-up when >1 FL was present. No patient had shown ≥60% plasma cells in bone marrow biopsy. Information on serum free light chain (SLFC) ratio was not available for this cohort. For 19 patients, genetic tests from bone marrow biopsy at initial wb-MRI were available; however, this subgroup was too small to run subgroup analysis to correlate MRI findings with genetic patterns.

### 2.4. Statistical Analysis

The primary clinical endpoint was time to progression (TTP) for analyzing risk of progression, defined as time between the first MRI in which the imaging parameter was assessed and progression. Deaths without prior progression to symptomatic myeloma were considered as a competing event. To assess the correlation between longitudinally analyzed imaging parameters and the risk of progression, cause-specific Cox regression with imaging biomarkers as time-dependent variables were used. To improve comparability of hazard ratios (HRs) for quantitative markers, all MRI parameters were standardized so that HRs report the change per standard deviation. For comparison of prognostic discrimination between different biomarkers, Harrell’s c-index was used. For assessment of correlations between imaging and continuous lab parameters, Spearman’s correlation coefficient was used, and for categorical parameters, the Wilcoxon test was used. A linear mixed model was used to assess the longitudinal association between hemoglobin and imaging parameters, as well as for the association between appearance/presence of OL and imaging parameters accounting for repeated measurements. Analysis was performed with software R 3.6 [38].

## 3. Results

### 3.1. Spatial Heterogeneity in Local Growth Dynamics

From this cohort of 60 patients with longitudinal imaging and overall 199 wb-MRIs, we analyzed FL volumes and local growth dynamics of 141 different FLs in 26 patients. Twenty-one patients had a total of 74 FLs at baseline. During follow-up, 67 new FLs appeared in 15 different patients.

**Figure 1:**
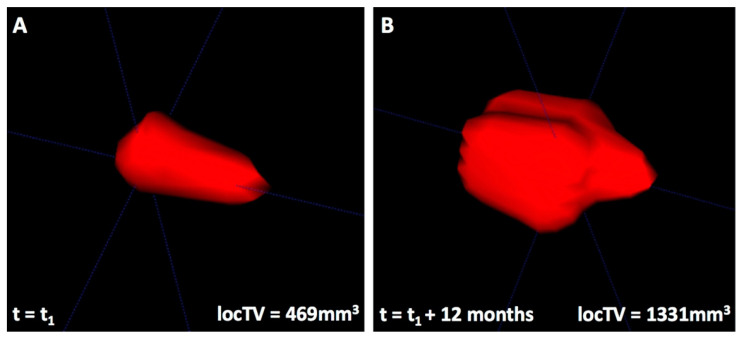
3D spatial tumor development of a focal lesion over time (t) in a female patient (55 years at t = t_1_). Segmented focal lesion images were acquired in a T2-weighted sequence and visualized by ITK-SNAP 3D-Viewer with relative scale and orientation. The presented focal lesion is located in the sacrum and shows a volume (V) of 469 mm^3^ in (**A**) and of 1331 mm^3^ 12 months later in (**B**), corresponding to a speed of growth of 2.36 mm^3^/day.

An example of the 3D spatial development of a FL is shown in Figure 1. In 12 of the 21 patients with ≥2 FL, we observed a difference of at least a factor 10 in local speed of growth between different FLs at one time-point during the follow-up period; 15 showed at least a factor 5 difference, and 19 at least a factor 3 difference, indicating that the wide majority of patients with more than 1 FL showed substantial differences of growth dynamics between tumor sites during their development to symptomatic MM. 

We performed a subgroup analysis excluding patients who had shown a concomitant plasma cell tumor. Within the remaining 52 patients, 19 patients showed a total of 61 different FLs during follow-up. Sixteen patients had a total of 39 FLs at baseline, and 22 new FLs appeared in 9 patients during follow-up. Of 14 patients who showed ≥2 FL during follow-up, in 7 patients we observed a difference of at least a factor 10 in local speed of growth between different FLs at one time-point; in 10 and 11 patients, a difference of at least factor 5 and factor 3 was observed.

### 3.2. Predictive Value of Focal Lesion Size and Growth Dynamic and Diffuse Infiltration Score

After demonstrating differences in local growth dynamics, the predictive value of FL size and growth dynamics as well as diffuse infiltration score regarding progression to symptomatic MM (defined by CRAB-criteria [25]) was investigated. All investigated MRI parameters showed significant correlations to development of symptomatic MM, as shown in Table 1. The speed of growth of the largest FL and the speed of growth of the fastest growing FL showed the best prognostic discrimination (c-index 0.745 and 0.749), both outperforming the size of the largest FL (c-index 0.715). The DIS and its development (DIS dynamic) showed inferior prognostic discrimination (both c-index 0.673).

The prognostic significance of the volume of the largest FL and the speed of growth of the fastest growing FL are independent from initial m-protein levels, as shown in Table 2.

There was no significant correlation between the tumor mass organized as FLs (represented by the sum of all FL volumes) and the tumor mass organized as diffuse infiltration (represented by the DIS; *p* = 0.86). Furthermore, the dynamic of the tumor mass organized as focal lesions and the dynamic of the tumor load organized as diffuse infiltration did not correlate (*p* = 0.81).

A subgroup analysis after exclusion of patients who had shown a concomitant plasma cell tumor and exclusion of measurements with >1 FL in wb-MRI was performed, including 40 SMM patients with at least 2 wb-MRIs. The size of the single FL was still a significant risk factor for progression (c-index = 0.576, HR = 1.43, *p* = 0.006). The speed of growth (SOG) of the single FL showed borderline statistical significance in this smaller subgroup (c-index = 0.578, HR = 9.94, *p* = 0.055 and c-index = 0.603, HR = 9.60, *p* = 0.054, respectively). The diffuse infiltration score did not show prognostic significance in this analysis (c-index = 0.558, HR = 1.19, *p* = 0.376).

### 3.3. Correlation of Focal Lesion Size and Growth Dynamic with Presence/Appearance of Corresponding Osteolytic Lesions in CT Imaging

**Figure 2:**
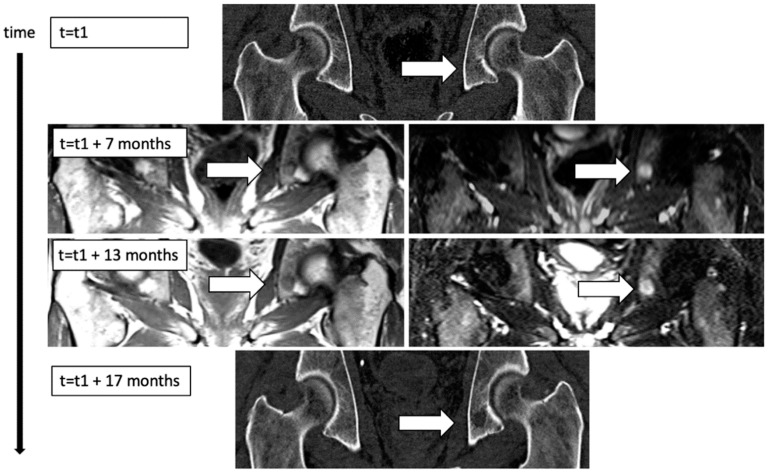
Development of a focal lesion in MRI over time with appearance of a new, corresponding osteolytic lesion in computed tomography (CT) imaging. At initial CT-imaging (t1), trabecular Scheme 1219. mm^3^, upper left (T1w tse) and right (T2w stir)), which then showed a three-dimensional growth dynamic of 3.37 mm^3^/day for 6 months to reach a volume of 1833 mm^3^ (t_2_ = 13 months, lower left and right). Four months later, the next CT imaging was performed and revealed an osteolytic lesion (t_3_ = 17 months, volume = 1022 mm^3^, lower image) in the corresponding position.

Next, it was investigated whether there was a correlation between characteristics of FLs in MRI with the presence/appearance of a corresponding OL in a near-term CT. FLs showing a corresponding OL in CT within 2 years from MRI assessment had both a significantly larger volume (mean volume 1210 mm^3^, *n* = 53 vs. 533 mm^3^, *n* = 37; *p* = 0.016) and a significantly higher mean speed of growth (mean speed of growth 3.12 mm^3^/day vs. 1.58 mm^3^/day, *p* = 0.022) than FLs which did not show a corresponding OL in CT within 2 years from MRI assessment. Figure 2 displays an example of a FL which showed development of a corresponding OL in CT over time.

While correlations of the volume and speed of growth of the FLs to the existence/appearance of OL were highly significant, the discriminative ability of both parameters was limited (Aera under the receiver operating curve (ROC AUC) 0.622 and 0.575, respectively). 

For simplicity, we decided to use 1000 mm^3^ as a cut-off for FL volumes, representing for example an FL measuring 1 cm by 1 cm by 1 cm. Of 71 FLs ≤1000 mm^3^, for only 37 FLs (52.1%), an OL was detected in a CT/PET-CT within 2 years, while for 16 out of 19 FLs (84.2%) >1000 mm^3^, an OL was detected in a CT/PET-CT within 2 years (*p* = 0.01). Of note, patients with a concomitant plasma cell tumor which had been treated with local radiation therapy before inclusion to MRI follow-up had a significantly higher risk of progression (*p* < 0.001) and had a higher proportion of patients developing OL (3 out of 8 (38%) vs. 9 out of 52 (17%)) compared to patients without a concomitant plasma cell tumor.

After exclusion of patients who presented a concomitant plasma cell tumor or >1 FL in wb-MRI, the subgroup for which wb-MRIs with exactly one FL and CT follow-up within 2 years was available contained only 3 patients. Of note, both FLs >1000 mm^3^ revealed a corresponding OL in a CT scan within 2 years, while one FL ≤1000 mm^3^ did not show a corresponding OL in a CT scan within 2 years.

### 3.4. Correlation of Diffuse Infiltration with Decrease in Hemoglobin

**Figure 3:**
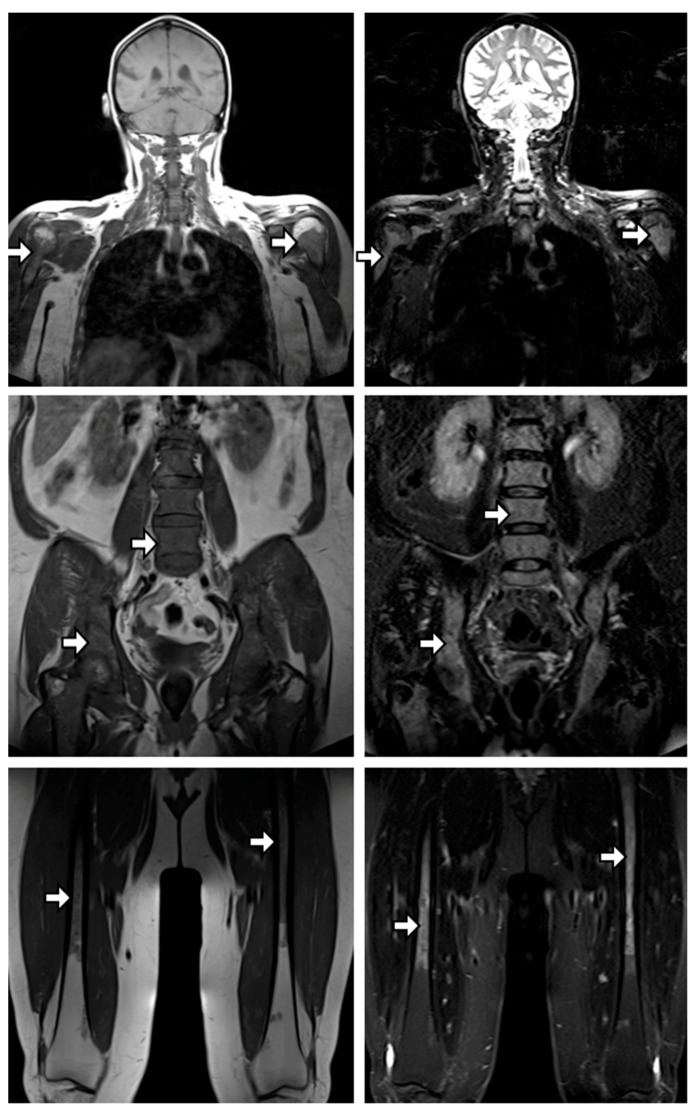
Ubiquitous severe diffuse infiltration. Coronal T1 tse and T2w stir images from a 57-year-old female patient at date of progression due to anemia (hemoglobin = 9.3 g/dL) are displayed. Over the course of the smoldering multiple myeloma (SMM) stage, this patient developed increasing diffuse infiltration, at progression showing severe infiltration in all parts of the skeleton including humeri (upper), spine and pelvis (middle), and femora (lower), with approximately muscle-/disc-isointense bone marrow intensities in T1w tse and marked hyperintense signal in T2w stir images, resulting in the maximal diffuse infiltration score of 24.

There was a significant correlation between DIS and decrease of hemoglobin level (*p* < 0.001). This correlation remained significant in the subgroup analysis after exclusion of patients with concomitant plasma cell tumor or >1 FL in wb-MRI (*p* = 0.003). Figure 3 shows a patient with ubiquitous severe diffuse infiltration who progressed in a timely manner to the presented MRI due to anemia (hemoglobin = 9.3 g/dL).

When investigating connections between baseline laboratory parameters (serum m-protein, bone marrow plasma cell percentage, creatinine, calcium, platelets, leucocytes, albumin, beta-2-microglobulin) and size and growth of isolated FLs, no significant correlations were found (all *p* > 0.05). Additionally, there were no significant correlations observed between development of local tumor site characteristics and development of laboratory parameters (all *p* > 0.05).

To investigate whether the correlation of DIS to hemoglobin is influenced by a concomitant increase in tumor mass organized as FLs, a multivariate analysis accounting for DIS, total tumor volume (sum of all FL volumes), dynamic of total tumor volume over time, and time was performed. DIS remained the only factor which was still significantly correlated to decrease in hemoglobin, as shown in Table 3.

## 4. Discussion

In this study, it was investigated whether MRI findings can help to better understand the development of monoclonal plasma cells disorders, and especially to understand when and how end organ damage occurs. This retrospective long-term analysis of longitudinal wb-MRI in untreated SMM patients reveals spatial heterogeneity in growth dynamics between different FLs, and the prognostic value of size and speed of growth of isolated FLs for both local and overall disease progression. Additionally, it indicates close connections between MRI findings and clinical progression patterns.

### 4.1. Spatial Heterogeneity in Growth Dynamics between Different Focal Lesions

Genetic heterogeneity between different FLs in MM patients has recently been reported [3]. As different local clones might differ regarding cell division rates and invasiveness and consequently lead to different growth dynamics between different FLs, it was investigated whether different growth dynamics between different FLs can be observed by MRI in untreated SMM patients. Indeed, it was found that there is marked heterogeneity in growth dynamics between different FLs in the majority of patients. It seems reasonable that genetic heterogeneity as reported in MM [3] is partially responsible for this finding. However, the growth dynamics of FLs are influenced by multiple factors; for example, local growth obstructions like adjacent cortical bone and difference in trabecular bone structure between different bone marrow sites. Therefore, no definite conclusion can be drawn whether or in which amount differences in local tumor genetics are responsible for the observed heterogeneity in growth dynamics. However, it can be described that spatial growth heterogeneity is observed in a considerable proportion of untreated SMM patients, and the background for this finding should be investigated by future molecular studies.

### 4.2. Predictive Value of FL Characteristics and Quantification of Diffuse Infiltration

Several publications have reported on the influence of the number of FLs on progression in monoclonal gammopathy of undetermined significance [6], in SMM [5,7,8] and in symptomatic MM [39,40,41]. Additionally, the general dynamics of MRI findings in SMM [19] and in symptomatic MM before and after treatment [39,41,42] have been shown to be of prognostic impact. However, only recently it was reported that the size of FLs in newly diagnosed MM patients is of importance for prognosis in these patients [18]. The results of our study demonstrate the influence of size and growth dynamic of individual FLs on progression in untreated SMM patients, with the growth dynamic of the fastest growing FL showing higher predictive value than the size of the largest FL. Using the DIS to more precisely quantify diffuse infiltration by integrating both local severity and overall extension of diffuse infiltration gave significant correlations between DIS/DIS dynamic and risk of progression. However, even with the effortful quantification performed in this study, the prognostic value was markedly lower compared to the number of FLs. It is noteworthy that both extent and dynamic of tumor mass organized as FLs and tumor mass organized as diffuse infiltration showed no correlations, indicating the independence of these two MRI findings from each other. 

Despite the small sample size of patients with 0 or 1 FL, the subgroup analysis indicates that also under the current definition of SMM, size and growth dynamic of a single lesion are connected to the risk of progression.

### 4.3. Connection between MRI Findings and Clinical Progression Patterns

While multiple studies have compared diagnostic performance between MRI and CT/X-rays to detect bone involvement in MM [21,22], to our best knowledge, no correlation between FL characteristics and presence or appearance of OL has been described. This study reveals that the size and growth dynamics of FLs significantly correlate with the presence/appearance of OL in near-term CT. Despite the high statistical significance, the AUC showed that the discriminative power of size/growth dynamics of FLs for the presence/appearance of OLs was limited. Since the integrity of mineralized bone is not depicted by MRI directly, the link between FL characteristics and OLs is not direct imaging of bone destruction. It rather reflects a probability that local myeloma cells in the imaged FL have acquired certain properties which lead to local destruction of the mineralized bone, even though the exact underlying molecular processes are not fully understood to date. Further reasons for the limited discriminative ability are the inter-location and inter-patient heterogeneity of physiological trabecular structure, which influence both origination and detection of OLs, and the limitations of this study as mentioned below. Recent IMWG guidelines now recommend alternating CT and MR imaging every 6 months for SMM patients with solitary FLs [43], and the result of this study that around 50% of FL ≤1 cm^3^ and around 80% of FLs >1 cm^3^ revealed OL in CT within 2 years supports their recommendation to use intermittent CT for follow-up of SMM patients with FLs to detect bone destruction. In a recent study, longitudinal low-dose wb-CT in SMM has been demonstrated to detect early bone progression due to OL >5 mm [37]. Future studies with such combined low-dose wb-CT- and wb-MRI-follow-up in SMM would allow refinement of our results and might allow defining size or growth dynamics of FLs as biomarkers to more exactly predict development of OLs. 

In symptomatic MM, an association between severe anemia and diffuse infiltration has been described [23,24]. The recently introduced “Myeloma Response Assessment and Diagnosis System” (MY-RADS) suggests to integrate diffuse infiltration from different regions into a total burden score [44]. Here, we introduced the DIS to quantify both severity and extent of overall diffuse infiltration in SMM patients, which revealed a significant correlation with decrease in hemoglobin.

OLs can be considered as localized events of a systemic disease, and consequently could be encountered with additional localized treatment. OLs account for the wide majority of progressions from SMM to MM [19], and around 90% of MM patients develop OLs throughout the course of their disease [20], making OL the main factor for morbidity in MM. While diverse parameters from imaging, laboratory values, or genetic aberrations have been proposed for risk stratification in SMM, these stratify in general high-risk vs. low-risk groups. In contrast, the results from this work indicate relationships between MRI findings and clinical progression patterns—certain FLs correlate with near-term bone destruction at this site, while diffuse infiltration is connected to reduction of hemoglobin. Therefore, size and growth dynamics of FLs might serve as biomarkers to predict upcoming OLs in the future, with the ultimate goal to prevent the development of OLs with additional, early, localized therapies of such FLs. This should be further investigated in patients with SMM according to the recent guidelines, as well as in patients with MM after systemic therapy.

### 4.4. Limitations

While this dataset with long-term longitudinal wb-MRI in SMM patients offered a valuable possibility to investigate MRI findings and correlating clinical progression patterns before initiation of systemic therapy, it is a limitation regarding immediate translation of our results into clinical practice that patients with >1 FL are now considered to have MM and are offered systemic therapy. Therefore, we performed subgroup analyses and found that even in patients with 0 or 1 FL, the size of the FL is connected to the risk of progression to MM. Growth dynamic showed only borderline significance in the small subset, while the diffuse infiltration score and its development were no longer a significant risk factors. While the subgroup for the existence/appearance of OL was too small for statistical testing, we described three cases exemplifying that our results might also translate to the current clinical situation. Larger studies in SMM patients according to the current definition and in patients after therapy should be performed in order to investigate whether the appearance of OL can be predicted by FL size and/or dynamic in these situations. The retrospective character and the fact that MR and CT imaging did not follow an exact predefined scheme with homogenous intervals between the scans are limitations of this study. Despite the high number of 199 wb-MRIs contributing information to this work, it is a limitation that these originate from only 60 patients. CT imaging was not performed simultaneously to every wb-MRI so that it cannot exactly be stated whether an OL has preexisted or appeared after the FL characteristic was measured, which we acknowledge using the phrase “presence/appearance of OL”. While it is a limitation that eight CTs and the three PET-CTs had different CT scan parameters than the dedicated wb low-dose CT protocol which was used for myeloma imaging in the majority of cases, using only CT/PET-CT and excluding X-ray for detection of corresponding OLs provides high data quality regarding detection of OLs.

## 5. Conclusions

Local growth dynamics of FLs differ substantially between different sites in the majority of former SMM patients. They correlate with risk of progression and local bone destruction at the respective site. The correlations between FL size/growth and OL, and between diffuse infiltration and hemoglobin decrease, indicate connections between MRI findings and clinical progression patterns in SMM patients. This supports the IMWG recommendation to perform intermittent CT imaging for follow-up in SMM patients with FLs to detect bone destruction at an early stage. Size and growth dynamics of FLs have the potential to become biomarkers to predict upcoming OL, which should be investigated by further studies on patients with SMM and patients after systemic therapy.

## Figures and Tables

**Table 1:** Predictive value of size and speed of growth (SOG) of focal lesions (FL) and diffuse infiltration regarding progression to symptomatic MM (multiple myeloma). Significant findings are given in bold and italic letters.

MRI-Based Biomarker	Harrelߣs c-index	Hazard Ratio	*p*-Value
Volume of the largest FL	0.715	1.48	***0.001***
SOG of the largest FL	0.745	1.97	***0.002***
SOG of the fastest growing FL	0.749	1.99	***0.003***
Diffuse infiltration score	0.673	1.69	***0.014***
Diffuse infiltration score—dynamic	0.673	1.77	***<0.001***

**Table 2:** Multivariate analysis of magnetic resonance imaging (MRI) parameters and baseline serum m-protein. SOG: speed of growth; FL: focal lesion. Significant findings are given in bold and italic letters.

Multivariate Analysis	Hazard Ratio	*p*-Value
M-protein	1.10	***0.002***
Volume of the largest FL	1.90	***0.009***
M-protein	1.11	***<0.001***
SOG of the fastest growing FL	2.15	***0.012***
M-protein	1.15	***0.01***
Diffuse infiltration score	0.86	0.70
M-protein	1.12	***0.02***
Dynamic of diffuse infiltration score	1.35	0.28

**Table 3:** Multivariate linear mixed model on hemoglobin. DIS: diffuse infiltration score; log_TTV: log-transformed total tumor volume summing up the volumes of all focal lesions; log_TTV-dynamic: log-transformed value of the dynamic of the total tumor volume over time; beta: model estimate. Significant findings are given in bold and italic numbers.

Multivariate Model	Beta	*p*-Value
DIS	−0.19	***<0.001***
log_TTV	−0.05	0.59
time	−0.00	0.18
DIS	−0.18	***<0.001***
log_SOG	0.55	0.79
time	−0.01	0.14

## Data Availability

Data not openly available due to privacy/ethical/legal restrictions (general data protection regulation of the EU) due to the retrospective character of the study with waiver of informed consent, granted by the Institutional Ethics Committee of the Medical Faculty of the Heidelberg University, Germany (S-511/2016).

## References

[1] Neben K., Jauch A., Hielscher T., Hillengass J., Lehners N., Seckinger A., Granzow M., Raab M.S., Ho A.D., Goldschmidt H. (2013). Progression in smoldering myeloma is independently determined by the chromosomal abnormalities del(17p), t(4;14), gain 1q, hyperdiploidy, and tumor load. J. Clin. Oncol..

[2] Fonseca R., Bergsagel P.L., Drach J., Shaughnessy J., Gutierrez N., Stewart A.K., Morgan G., Van Ness B., Chesi M., Minvielle S. (2009). International Myeloma Working Group molecular classification of multiple myeloma: Spotlight review. Leukemia.

[3] Rasche L., Chavan S.S., Stephens O.W., Patel P.H., Tytarenko R., Ashby C., Bauer M., Stein C., Deshpande S., Wardell C. (2017). Spatial genomic heterogeneity in multiple myeloma revealed by multi-region sequencing. Nat. Commun..

[4] Kyle R.A., Remstein E.D., Therneau T.M., Dispenzieri A., Kurtin P.J., Hodnefield J.M., Larson D.R., Plevak M.F., Jelinek D.F., Fonseca R. (2007). Clinical Course and Prognosis of Smoldering (Asymptomatic) Multiple Myeloma. N. Engl. J. Med..

[5] Hillengass J., Fechtner K., Weber M.-A., Bäuerle T., Ayyaz S., Heiss C., Hielscher T., Moehler T.M., Egerer G., Neben K. (2010). Prognostic Significance of Focal Lesions in Whole-Body Magnetic Resonance Imaging in Patients With Asymptomatic Multiple Myeloma. J. Clin. Oncol..

[6] Hillengass J., Weber M.A., Kilk K., Listl K., Wagner-Gund B., Hillengass M., Hielscher T., Farid A., Neben K., Delorme S. (2014). Prognostic significance of whole-body MRI in patients with monoclonal gammopathy of undetermined significance. Leukemia.

[7] Kastritis E., Moulopoulos L.A., Terpos E., Koutoulidis V., Dimopoulos M.A. (2014). The prognostic importance of the presence of more than one focal lesion in spine MRI of patients with asymptomatic ( smoldering ) multiple myeloma. Leukemia.

[8] Dhodapkar M.V., Sexton R., Waheed S., Usmani S., Papanikolaou X., Nair B., Petty N., Shaughnessy J.D.J., Hoering A., Crowley J. (2014). Clinical, genomic, and imaging predictors of myeloma progression from asymptomatic monoclonal gammopathies (SWOG S0120). Blood.

[9] Dispenzieri A., Kyle R.A., Katzmann J.A., Therneau T.M., Larson D., Benson J., Clark R.J., Melton L.J., Gertz M.A., Kumar S.K. (2008). Immunoglobulin free light chain ratio is an independent risk factor for progression of smoldering (asymptomatic) multiple myeloma. Blood.

[10] Kastritis E., Terpos E., Moulopoulos L., Spyropoulou-Vlachou M., Kanellias N., Eleftherakis-Papaiakovou E., Gkotzamanidou M., Migkou M., Gavriatopoulou M., Roussou M. (2013). Extensive bone marrow infiltration and abnormal free light chain ratio identifies patients with asymptomatic myeloma at high risk for progression to symptomatic disease. Leukemia.

[11] Larsen J.T., Kumar S.K., Dispenzieri A., Kyle R.A., Katzmann J.A., Rajkumar S.V. (2013). Serum free light chain ratio as a biomarker for high-risk smoldering multiple myeloma. Leukemia.

[12] Waxman A.J., Mick R., Garfall A.L., Cohen A., Vogl D.T., Stadtmauer E.A., Weiss B.M. (2015). Classifying ultra-high risk smoldering myeloma. Leukemia.

[13] Rajkumar S.V., Larson D., Kyle R.A. (2011). Diagnosis of smoldering multiple myeloma. N. Engl. J. Med..

[14] Rajkumar S.V., Dimopoulos M.A., Palumbo A., Blade J., Merlini G., Mateos M.-V., Kumar S., Hillengass J., Kastritis E., Richardson P. (2014). International Myeloma Working Group updated criteria for the diagnosis of multiple myeloma. Lancet Oncol..

[15] Mateos M.-V., Hernandez M.-T., Giraldo P., de la Rubia J., de Arriba F., Lopez Corral L., Rosinol L., Paiva B., Palomera L., Bargay J. (2013). Lenalidomide plus dexamethasone for high-risk smoldering multiple myeloma. N. Engl. J. Med..

[16] Mateos M.-V., Hernandez M.-T., Giraldo P., de la Rubia J., de Arriba F., Corral L.L., Rosinol L., Paiva B., Palomera L., Bargay J. (2016). Lenalidomide plus dexamethasone versus observation in patients with high-risk smouldering multiple myeloma (QuiRedex): Long-term follow-up of a randomised, controlled, phase 3 trial. Lancet. Oncol..

[17] Lonial S., Jacobus S., Fonseca R., Weiss M., Kumar S., Orlowski R.Z., Kaufman J.L., Yacoub A.M., Buadi F.K., O’Brien T. (2019). Randomized Trial of Lenalidomide Versus Observation in Smoldering Multiple Myeloma. J. Clin. Oncol..

[18] Rasche L., Angtuaco E.J., Alpe T.L., Gershner G.H., McDonald J.E., Samant R.S., Kumar M., Van Hemert R., Epstein J., Deshpande S. (2018). The presence of large focal lesions is a strong independent prognostic factor in multiple myeloma. Blood.

[19] Merz M., Hielscher T., Wagner B., Sauer S., Shah S., Raab M.S., Jauch A., Neben K., Hose D., Egerer G. (2014). Predictive value of longitudinal whole-body magnetic resonance imaging in patients with smoldering multiple myeloma. Leukemia.

[20] Kyle R.A., Gertz M.A., Witzig T.E., Lust J.A., Lacy M.Q., Dispenzieri A., Fonseca R., Rajkumar S.V., Offord J.R., Larson D.R. (2003). Review of 1027 patients with newly diagnosed multiple myeloma. Mayo Clin. Proc..

[21] Caers J., Withofs N., Hillengass J., Simoni P., Zamagni E., Hustinx R., Beguin Y. (2014). The role of positron emission tomography-computed tomography and magnetic resonance imaging in diagnosis and follow up of multiple myeloma. Haematologica.

[22] Regelink J.C., Minnema M.C., Terpos E., Kamphuis M.H., Raijmakers P.G., van den Bos I.C.P., Heggelman B.G.F., Nievelstein R.J., Otten R.H.J., van Lammeren-Venema D. (2013). Comparison of modern and conventional imaging techniques in establishing multiple myeloma-related bone disease: A systematic review. Br. J. Haematol..

[23] Song M., Chung J., Lee J., Min C., Lee S., Shin D., Bae S., Hong J., Lee G., Lee I. (2014). Magnetic resonance imaging pattern of bone marrow involvement as a new predictive parameter of disease progression in newly diagnosed patients with multiple myeloma eligible for autologous stem cell transplantation. Br. J. Haematol..

[24] Moulopoulos L.A., Gika D., Anagnostopoulos A., Delasalle K., Weber D., Alexanian R., Dimopoulos M.A. (2005). Prognostic significance of magnetic resonance imaging of bone marrow in previously untreated patients with multiple myeloma. Ann. Oncol..

[25] The International Myeloma Working Group (2003). Criteria for the classification of monoclonal gammopathies, multiple myeloma and related disorders: A report of the International Myeloma Working Group. Br. J. Haematol..

[26] Durie B.G.M., Kyle R.A., Belch A., Bensinger W., Blade J., Boccadoro M., Child J.A., Comenzo R., Djulbegovic B., Fantl D. (2003). Myeloma management guidelines: A consensus report from the Scientific Advisors of the International Myeloma Foundation. Hematol. J.Off. J. Eur. Haematol. Assoc..

[27] Fechtner K., Hillengass J., Delorme S., Heiss C., Neben K., Goldschmidt H., Kauczor H.-U., Weber M.-A. (2010). Staging monoclonal plasma cell disease: Comparison of the Durie-Salmon and the Durie-Salmon PLUS staging systems. Radiology.

[28] Kloth J.K., Hillengass J., Listl K., Kilk K., Hielscher T., Landgren O., Delorme S., Goldschmidt H., Kauczor H.U., Weber M.A. (2014). Appearance of monoclonal plasma cell diseases in whole-body magnetic resonance imaging and correlation with parameters of disease activity. Int. J. Cancer.

[29] Wennmann M., Kintzelé L., Piraud M., Menze B.H., Hielscher T., Hofmanninger J., Wagner B., Kauczor H.-U., Merz M., Hillengass J. (2018). Volumetry based biomarker speed of growth: Quantifying the change of total tumor volume in whole-body magnetic resonance imaging over time improves risk stratification of smoldering multiple myeloma patients. Oncotarget.

[30] Gleeson T.G., Moriarty J., Shortt C.P., Gleeson J.P., Fitzpatrick P., Byrne B., McHugh J., O’Connell M., O’Gorman P., Eustace S.J. (2009). Accuracy of whole-body low-dose multidetector CT (WBLDCT) versus skeletal survey in the detection of myelomatous lesions, and correlation of disease distribution with whole-body MRI (WBMRI). Skeletal Radiol..

[31] Hillengass J., Moulopoulos L.A., Delorme S., Koutoulidis V., Mosebach J., Hielscher T., Drake M., Rajkumar S.V., Oestergaard B., Abildgaard N. (2017). Whole-body computed tomography versus conventional skeletal survey in patients with multiple myeloma: A study of the International Myeloma Working Group. Blood Cancer J..

[32] Wolf M.B., Murray F., Kilk K., Hillengass J., Delorme S., Heiss C., Neben K., Goldschmidt H., Kauczor H.U., Weber M.A. (2014). Sensitivity of whole-body CT and MRI versus projection radiography in the detection of osteolyses in patients with monoclonal plasma cell disease. Eur. J. Radiol..

[33] Piraud M., Wennmann M., Kintzelé L., Hillengass J., Keller U., Langs G., Weber M.-A., Menze B.H. (2019). Towards Quantitative Imaging Biomarkers of Tumor Dissemination: A Multi-scale Parametric Modeling of Multiple Myeloma. Med. Image Anal..

[34] Baur A., Stabler A., Nagel D., Lamerz R., Bartl R., Hiller E., Wendtner C., Bachner F., Reiser M. (2002). Magnetic resonance imaging as a supplement for the clinical staging system of Durie and Salmon?. Cancer.

[35] Poulton T.B., Murphy W.D., Duerk J.L., Chapek C.C., Feiglin D.H. (1993). Bone marrow reconversion in adults who are smokers: MR Imaging findings. Am. J. Roentgenol..

[36] Daldrup-Link H.E., Henning T., Link T.M. (2007). MR imaging of therapy-induced changes of bone marrow. Eur. Radiol..

[37] Gavriatopoulou M., Βoultadaki A., Koutoulidis V., Ntanasis-Stathopoulos I., Bourgioti C., Malandrakis P., Fotiou D., Migkou M., Kanellias N., Eleutherakis-Papaiakovou E. (2020). The Role of Low Dose Whole Body CT in the Detection of Progression of Patients with Smoldering Multiple Myeloma. Blood Cancer J..

[38] R Core Team (2020). R: A Language and Environment for Statistical Computing. https://www.R-project.org.

[39] Walker R., Barlogie B., Haessler J., Tricot G., Anaissie E., Shaughnessy J.D.J., Epstein J., van Hemert R., Erdem E., Hoering A. (2007). Magnetic resonance imaging in multiple myeloma: Diagnostic and clinical implications. J. Clin. Oncol..

[40] Mai E.K., Hielscher T., Kloth J.K., Merz M., Shah S., Raab M.S., Hillengass M., Wagner B., Jauch A., Hose D. (2015). A magnetic resonance imaging-based prognostic scoring system to predict outcome in transplant-eligible patients with multiple myeloma. Haematologica.

[41] Mosebach J., Shah S., Delorme S., Hielscher T., Goldschmidt H., Schlemmer H.-P., Schonland S., Hegenbart U., Hillengass J. (2018). Prognostic significance of tumor burden assessed by whole-body magnetic resonance imaging in multiple myeloma patients treated with allogeneic stem cell transplantation. Haematologica.

[42] Hillengass J., Ayyaz S., Kilk K., Weber M.-A., Hielscher T., Shah R., Hose D., Delorme S., Goldschmidt H., Neben K. (2012). Changes in magnetic resonance imaging before and after autologous stem cell transplantation correlate with response and survival in multiple myeloma. Haematologica.

[43] Hillengass J., Usmani S., Rajkumar S.V., Durie B.G.M., Mateos M.-V., Lonial S., Joao C., Anderson K.C., Garcia-Sanz R., Serra E.R. (2019). International myeloma working group consensus recommendations on imaging in monoclonal plasma cell disorders. Lancet. Oncol..

[44] Messiou C., Hillengass J., Delorme S., Lecouvet F.E., Moulopoulos L.A., Collins D.J., Blackledge M.D., Abildgaard N., Østergaard B., Schlemmer H.-P. (2019). Guidelines for Acquisition, Interpretation, and Reporting of Whole-Body MRI in Myeloma: Myeloma Response Assessment and Diagnosis System (MY-RADS). Radiology.

